# Crucial Dimensions of Human Altruism. Affective vs. Conceptual Factors Leading to Helping or Reinforcing Others

**DOI:** 10.3389/fpsyg.2016.00519

**Published:** 2016-04-13

**Authors:** Anna Szuster

**Affiliations:** Faculty of Psychology, University of WarsawWarsaw, Poland

**Keywords:** automatic interactions, affiliation, socialization, normative vs. moral standards

## Abstract

The aim of this article is to identify factors leading to favorable attitudes toward other people from different social categories. The parts of article reflect diverse levels of altruism regulation from primary affective responses to the environment, through social norms, to abstract moral concepts related to good and evil. The latter allow understanding of the perspective of other people (including those belonging to out-groups), acceptance of their values and engagement not only in helping behavior but also in supporting the development of others.

## The Many Facets of Altruism

When he first used the term “altruism” in a scientific context, August Comte could not have predicted the staggering career of the concept in humanities, as well as social and natural science, or the myriad alternative meanings it would spawn. For Comte, altruism denoted a type of social behavior expressed through living selflessly for the sake of others (Campel, 2006).

Today, the “well-being of others” remains the defining criterion of altruism, but the question of selflessness is debated. The dispute whether non-egoistic behavior is possible at all is the bone of contention (Cf. the Cialdini-Batson argument). But even the proponents of the “egoistic” view are using the term (Cialdini et al., 1981). One attempt to resolve the dilemma of selflessness was to look at costs, which are sometimes even interpreted as losses (Hamilton, 1964). The point is that the altruistic actor incurs significant, personal damage when acting for the sake of others. The import of these costs is supposed to invalidate suspected “egoistic” motivation for altruism thus defined. Nevertheless, with the exception of kin altruism (where the stakes can be as high as life or health), reciprocal altruism demands smaller sacrifices, being decidedly less spectacular (as clearly explained by Wilson, 1975). A more inclusive (Tooby and Cosmides, 1996) definition of altruism refers to all manner of actions for the benefit of others, some of which do not necessarily compromise the benefactor’s interests. It is manifested in assistance that carries no costs, in forging transient alliances, cooperation, sharing something of value with others, offering (anonymously) something.

Altruism is not limited to helping the disadvantaged. Other actions that tend to be equated with altruism include expressing positive emotions and empathy. Altruistic motivation is associated with commitment to values aimed at serving the best interest of others (Reykowski, 1989). The concept of altruism is so broad as to include species at the lower rungs of the evolutionary ladder. Some forms of cooperation in social insects as well as birds, are considered the most basic forms of altruism (Wilson, 1975).

The present paper deals with the broad definition of altruism, understood as the dispositional willingness to respond with positive emotions to others and to treat others, including strangers, sympathetically. Such sympathy not always translates to behavioral acts (this is the difference between the concept of “altruism” and that of “prosocial behavior”).

The purpose of the article is to present various possible internal dispositions not only innate to particular species or internalized during the course of socialization, but also shaped through values generated reflexively by subjects and standards determining the criteria of good and evil for others, the world, and even future generations. The paper focus on mechanisms forming direct or indirect premises of altruism.

## Reactive Nature of the Premises of Altruism: Primary, Automatic Interrelations with Others

The primary determinants of altruism are innate and universal and have been shaped by evolution (Wilson, 1975; Burnstein, 2005) and early childhood experiences (Warneken and Tomasello, 2006). Our insight into their specifics stems from developmental data and embodiment research. The basic ability to focus attention on others is reflected in automatically following the movement of another person’s eyes. *Homo sapiens* is the only species to have evolved gaze direction to be easily perceived by others (due to highly visible, white sclera) and only humans (already in infancy) use that information, which, in turn, facilitates the recognition of another person’s perspective – a skill important for the activation of a person’s altruistic engagement (Tomasello, 2014). Data reveal that early, non-verbal, bodily exchange, and communication lead to strong connections between the child and the environment. The abilities to mimic and to have synthonic reactions to the affective states of others have a special importance in the course a lifetime. The neural basis for these processes is known as “mirror neurons” (Iacoboni, 2005).

Mimicry as an automatic imitation of a partner’s behavior (this is known as “social glue”) connects people. Empirical findings have confirmed that mimicry leads to favorable treatment of the mimicker [e.g., giving larger tips to barmen who mimic patrons’ language and facial expressions: (van Baaren et al., 2003)]. The mechanism is reciprocal: the mimicker is more positively inclined toward the person mimicked. Mimicry increases the sense of interpersonal closeness, reciprocal similarity, and facilitates the flow of interaction. Mimicry increases the willingness to help, e.g., to spontaneously pick up coins dropped “by accident”. Help was offered both to the mimicker and to bystanders. One of the most common ways to perform an altruistic act is to donate to charity, either with your time, your money, or your material goods. The people being mimicked were more likely to donate to charities (van Baaren et al., 2004). Further experiments (Stel et al., 2008) have demonstrated the same mechanism for mimickers. In our studies we noticed increased donations to charity accompanied mimicry, even when the mimicker was in no way associated with the collection (Szuster, 2005).

Peripheral mechanisms triggered automatically during interaction also allow us to decode others’ facial expressions and to communicate our own affective states (Dimberg et al., 2000). The primary, automatic mechanism of affective contagion (e.g., as in the experiment where infants exposed to crying responded by crying themselves – see Simner, 1971) is considered to be the base for subsequent emotional empathy. It is reactive, instinctive and it triggers a circular reaction (Hoffman, 2000), in which a person’s own state is no longer distinguishable from the suffering of another person. Empathy, based on the affective mechanism (Hoffman, 1975, 2000), results from the ability to respond automatically to other people expressing emotions. Its distinctive feature is the psychological separation of the perceiver and recipient of someone’s emotion from the individual experiencing that emotion. The active nature of this process creates a new quality of altruism responding, introducing certain “filters” between one’s own and other people’s emotional states (Baron-Cohen, 2011) and the harm and well-being of others is not equivalent to one’s own.

Numerous data confirm the relationship between empathy and altruism, cooperation and just distribution of goods (Hoffman, 1975, 2000; Eisenberg and Morris, 2001). Priming with empathy was shown to increase sensitivity to the needs of others and to promote positive attitudes toward members of stigmatized groups: AIDS sufferers, homeless people, criminals (Batson, 1997) and minorities (Vescio et al., 2003). Our own findings on cyberbullying supported the effectiveness of empathy activation in virtual reality. Priming with empathy reduced the frequency by adolescents sending comments that compromised their peers. However, these effects were transient. Empathy was successful in reducing cyberbullying only when triggered immediately before the measurement of the behavior (Barlińska et al., 2013).

Empathy does not always strengthen altruistic behaviors. This is particularly true of the affective empathy. Discomfort generated as a reaction to the suffering of another person produces the reaction of avoidance, depreciation of the other person (the concept of emphatic anger – Hoffman, 2000) and even an attack or an act of aggression. The latter occurs when the sharing of emotions concerns aggression. In short, the specifics of the primary mechanisms of altruism are their reflexive, involuntary, and automatic nature. These mechanisms have limitations. They require direct contact. However, the memory of such experiences enables individuals (through mental images) to also respond with empathy to other persons, irrespective of how distant in space and time they may be (Hoffman, 2000).

## Socialization as the Fundament of Normative Altruism

The external world triggers reflexive approach or avoidance responses. Seeking interaction with others is fostered by the biological craving for sensory stimulation, without which it is impossible to maintain homeostasis. Mere exposure to neutral stimuli (i.e., those that carry no threat) later trigger elementary positive responses (Zajonc, 1968). Lasting contact encourages increasing familiarity with the environment and enhanced involuntary attachment. However, the main social foundations of altruism are rooted in seeking contact with others in order to obtain reinforcement for the purposes of safety, the sense of belonging and affiliation (Schachter, 1959; Baumeister and Leary, 1995). A characteristic aspect for this category of altruism is that the individual’s responses oriented towards other people are instrumental to that subject’s own needs. Reinforcements provide a platform for creating a psychological dependence, which induces the subject to meet external expectations (despite punishment) and to conform to social norms including those that require altruism and condemn egoism.

Social norms are instilled through the educational process, whereby social actors (parents, teachers, peers, in-group members) verbalize their expectations toward individuals. However, socialization is also affected by a wide variety of non-verbal factors operating in the social environment (Hoffman, 2000). They include ways of responding to others, feeling sympathetic, or antipathetic toward them. These messages may take the form of microexpressions, and other subtle reflections of behavior (e.g., distance, physical, and psychological closeness). Thus, some attitudes, both explicit, and implicit are formed as a result of socialization (Greenwald and Banaji, 1995). The environment offers behavioral patterns (algorithms) approved in a given community and the culture prevalent in that community. The subject repeats them with no need for verbalization. Thus morality, including the imperative of being helpful, can have an intuitive nature, associated with the sense of good without the ability to understand the essence of the principles of acting properly (Jarymowicz, 2015). Gradually, the range of individual attitudes depends on what is typical of the groups one belongs to. Younger children spontaneously express aversion to others, while older children try to hide it (cf. the *Pollyanna hypothesis:* Drozda-Senkowska, 1990). Internalized social norms become the source for standards of “normal states” (Reykowski, 1989). Failure to meet them becomes a reason for internal punishments. More or less conscious realization that helping others is highly valued in society makes that strategy a norm whose fulfillment provides gratification and maintains or enhances positive mood, and reduces the negative one. A series of experiments proved that following the helpfulness norm can be instrumental in the affective well-being of the individual (Schwartz, 1977; Staub, 1979; Cialdini et al., 1981). This, in turn, perpetuates the motivation to engage in helping others.

Social norms, associated (unconsciously or reflexively) with self-esteem become personal, which significantly enhances their control over behavior (Schwartz, 1977). There have been numerous empirical findings confirming the regulatory role of self-esteem in inspiring altruistic behavior (Berkowitz and Daniels, 1963; Karyłowski, 1982; Schwartz and Howard, 1982). This rule also concerns volunteers who, apart from having higher self-esteem, are happier, more optimistic and generally more content with their lives (Piliavin, 2003).

People are usually not consciously aware of these relationships. Breaking a norm (punishing partner’s mistakes with electric shocks in an experiment, or lying) increased helpful behavior in the next task both towards the same person in a different context (Berkowitz, 1972). In our research, feedback about participants’ high social competence increased the amount of memorized data about a person in need and the number of ideas generated for helping (Szuster, 2005).

In summary: in the course of social development, human behaviors, including the ones which do not benefit the helper, are regulated by certain norms. People learn quickly that helping others is a highly regarded value. Compliance of behavior with the norm stabilizes affective well-being and is also a means of maintaining a positive self-image.

## Concepts, Reflections, and Moral Values as Bases for Altruism

As a result of socialization, the individual gains knowledge (intuitive or verbalized) of what is right and wrong – in terms of culture-specific prescriptions. The in-group standard contents of norms such as social responsibility (Berkowitz and Daniels, 1963), sharing and giving, justice or reciprocity (Walster et al., 1978) are all the factors leading to altruism. Their regulatory function is associated with anticipated reward or punishment (sometimes as delayed as salvation after death as a result of living according to religious teachings). Social approval or disapproval is the main basis of altruistic acts.

Such *normative altruism* is often limited to the norms of one’s reference group, which may be the source of altruism toward the in-group members, but at the same time a source of prejudice and hostility toward the out-group. The understanding of norms may be limited to one’s environment. In the experiments of Piliavin (see: Reykowski, 1979), donations to single mothers decreased as a function of physical distance: the most generous donations were given to mothers from the same town, the lowest to those living in another state. However, even a much smaller distance can mean that help is not extended to the person in need lying on the other side of the street (Staub, 1974).

Intellectual development contributes to increased cognitive complexity including self and social awareness (Piaget, 1965). The result is an improved understanding of the world and a multiplicity of perspectives. Intellectual skills pave the way for decentration: changing the cognitive perspective from egocentric to external. This, in turn, may lead to voluntary shifting and focusing of attention, and to conscious separation of one’s own and other people’s perspectives. Whether developmental or situational, shortage of decentration leads to egocentric biases in the perception of others and the world. The ability to take the perspective of others significantly increases the potential of human altruism (Batson et al., 1997). Understanding of social norms changes and the subject gradually becomes independent of algorithms imposed by the environment. Filled with individual content and bound to the Self, norms present one of the most common sources of Self standards: Ought or Ideal (Schwartz, 1977; Higgins, 1987). This leads to the emergence of new, internal imperatives for altruistic behavior.

Some mechanisms regulating altruistic behavior are stimulated when the so-called Ought Self standards (Higgins, 1987) are activated. In many cultures, obligations are limited to the requirement of helping others in trouble. This category of mechanisms should be treated separately from those that lead to supporting others in their development and improving their quality of life. They key to their comprehension appears to be the concept of the Ideal Self, related to moral values. While social norms and their internalization (in the form of the Ought Self standards) are essentially “local” and promote altruism predominantly toward the in-group, moral values are universal, and, as such, extend the scope of altruism to out-groups. When intellectual development allows reflective thinking and understanding of moral values, the subject becomes able to perceive the state of another person (regardless of social status and own interests). Cognitive complexity, and cognitive categories width (Pettigrew, 1979) facilitate openness to others, reduce prejudice, and promote positive attitudes. Jarymowicz (2015) argues that cognitive complexity becomes the determinant of a truly moral behavior if it leads to the so-called axiological complexity that is the understanding of the sense of such abstract concepts as *human rights* or *social justice*. The understanding of values expressed in abstract language requires the involvement of the intellect in reflective thinking. Once that is achieved, the standards of ideal states become the point of reference for the states of others, triggering altruistic motivation, and others are perceived as autonomous individuals having a value in and of themselves (Reykowski, 1979; Rutkowska and Szuster, 2011). Bolstered by ideals, altruistic involvement goes beyond restoring what a given society considers a balance (relieving hardship) and opens up to supporting development and expanding other people’s opportunities.

In short: the system of reflective thinking and evaluation alters the quality of human functioning (Deutsch and Strack, 2006; Jarymowicz and Imbir, 2015) with regard to understanding the meaning of abstract concepts referring to moral values in one’s own life. It increases the accessibility of various criteria of evaluating the situation of others, including what is generally understood as goodness. This, in turn, can cause the subject to get engaged more involved in further altruistic deeds.

## Conclusion

The article presents three group altruism-building mechanisms and focuses on the role of their biological, social and personal (conceptive, reflective) factors. They engage the body, as well as various parts of the human mind. Each type of mechanism generates reactions of a different nature.

The first basic mechanisms yield reactive and automatic reactions. These are universal, bodily mechanisms, which are the very basis of every type of reaction. Individual differentiation among them is slight. They constitute the “manufacturer’s equipment” of the person.

The second type of mechanism is the result of the process of learning the patterns of behaviors and understanding norms. They are the source of one’s engagement mostly in the well-being of one’s “in-group” members.

The last type of mechanism represents the outcome of intellectual involvement and reflective thinking. They lead to forming abstract evaluative concepts, which give rise to the emergence of entirely new standards of world evaluation.

The evolutionary processes to which man is subject are the very source of the mechanisms’ diversity. In the course of evolution, human beings have been equipped with automatic, affective mechanisms allowing them to approach others. This is the basis of altruism. However, the specificity of human altruism depends on the development of concepts enabling one to transcend the face-to-face context, to understand the perspective of others and to accept their values. Also, through intellect, anticipation of the interests of future generation is feasible. The differentiation between ought and ideal evaluative standards creates a chance to become involved in the development of another person, and not only to be content with charitable acts.

The knowledge about the sources of altruism mentioned above falls within the domain of biology and psychology alike. However, the emerging theories should not be treated as competitive. Described in terms of various scientific disciplines, the sources of altruism should be regarded as complementary theories aimed as explaining diverse forms of engagement in helping others. From the psychological point of view such an approach to altruism could be viewed as consistent with the dual mind theories developed by many researchers (Deutsch and Strack, 2006; Jarymowicz and Imbir, 2015).

## Author Contributions

My role in creating the manuscript included: (1) Substantial contributions to the conception or design of the work; and (2) Drafting the work or revising it critically for important intellectual content; and (3) Final approval of the version to be published; and (4) Agreement to be accountable for all aspects of the work in ensuring that questions related to the accuracy or integrity of any part of the work are appropriately investigated and resolved.

## Conflict of Interest Statement

The author declares that the research was conducted in the absence of any commercial or financial relationships that could be construed as a potential conflict of interest.
